# Micro: a useful and simpler tool to measure the magnitude of scoliosis curves on x-rays

**DOI:** 10.1186/1748-7161-8-S1-O26

**Published:** 2013-06-03

**Authors:** M Romano, F Zaina, S Donzelli, S Negrini

**Affiliations:** 1 ISICO (Italian Scientific Spine Institute), Milan, Italy; 2 University of Brescia, Brescia, Italy; 3 IRCCS Don Gnocchi, Milan, Italy

## Background

The gold standard method for the measurement of the magnitude of a scoliotic curve on x-rays is the Cobb, or Cotrel, method. With these methods, it is necessary to draw on the radiograph a series of segments and to measure with a protractor the angle formed by the intersection of two of them.

## Aim

The purpose of this study is to describe a different method for the measurement of the scoliosis angles on x-rays (Micro method), and to verify the concordance with the gold standard.

## Methods

The Micro method is based on the use of a Bunnell scoliometer. The sum of the inclinations of the vertebrae limiting the curve determines the angle of the curve. Three experienced physicians measured 30 X-rays with the Micro method and the Cotrel method. The extent of correlation between the two measurements was performed using the Bland Altman plots.

## Results

The differences between the 90 measurements made with two different methods has never exceeded the commonly accepted measurement error (+ / -5 °), while the average of these differences was 0.02 ° (St. Dev. 1.89). The normal distribution demonstrates the high degree of correlation between the two methods of measurement. To evaluate the degree of consistency among the 30 measurements performed by the three physicians with the two different methods of measurement, the average discrepancy in the following format was calculated:

Mean discrepancy Cotrel Micro;

Physician 1 vs Physician 2 (1.36° vs 0.16°)

Physician 1 vs Physician 3 (0.16° vs 0.13°)

Physician 2 vs Physician 3 (12° vs 0.03°)

All comparisons show a greater degree of agreement between the measurements performed using the Micro method compared to the gold standard.

## Conclusion

For the measurement of a scoliotic curve on a conventional x-ray, the results of this study demonstrate a high reliability of the Micro method compared with the gold standard. The measurement with the Micro method is faster, and easy to obtain, even for non-experienced operators. The use of the scoliometer, in fact, reduces the errors of the correct identification of the vertebrae limiting the curve.
